# Comparison of survival outcomes between primary and secondary muscle-invasive bladder cancer: An updated meta-analysis

**DOI:** 10.7150/ijms.49228

**Published:** 2021-01-01

**Authors:** Xiaonan Zheng, Shi Qiu, Lu Yang, Qiang Wei

**Affiliations:** 1 Department of Urology, Institute of Urology, West China Hospital, Sichuan University, Chengdu, Sichuan. P.R. China.; 2 Center of Biomedical big data, West China Hospital, Sichuan University, Chengdu, Sichuan, P.R. China.

**Keywords:** primary muscle-invasive bladder cancer, secondary muscle-invasive bladder cancer, initial diagnosis, radical cystectomy, cancer-specific mortality, overall mortality, recurrence

## Abstract

**Objective:** Studies have showed that different follow-up starting points might potentially impact the comparison between primary (PMIBC) and secondary muscle-invasive bladder cancer (SMIBC), but the only previous meta-analysis did not differentiate the follow-up starting points of included studies. With more trials published, we aim to update the meta-analysis comparing PMIBC and SMIBC.

**Methods:** PubMed, Embase, Cochrane Library and ClinicalTrial.gov. systematically searched. Literatures comparing the survival outcomes of PMIBC and SMIBC were selected. Outcomes of cancer-specific mortality (CSM), overall mortality (OM) and recurrence-free survival (RFS) were pooled and grouped based on the starting point of follow-up (after initial diagnosis or radical cystectomy (RC)). Newcastle-Ottawa Scale (NOS) and funnel plot were employed to assess the study quality and publication bias, respectively.

**Results:** A total of 17 high-quality studies were selected, with 5558 patients aged from 59.8 to 72.7 (mean value) involved. The male-to-female ratio was roughly 4:1 (4390/1124). SMIBC had lower risk of CSM after initial diagnosis (HR 0.81, 95%CI 0.67-0.98, *P=*0.03, I^2^=70%), but higher risk of CSM after RC (HR 1.45, 95%CI 1.27-1.65, *P<*0.00001, I^2^=64%). In terms of OM and recurrence, outcomes were pooled only after RC, which both turned out to be higher for SMIBC (OM: HR 1.50, 95%CI 1.30-1.73, *P<*0.00001, I^2^=0%; Recurrence: HR 1.66, 95%CI 1.36-2.02, *P<*0.00001, I^2^=48%). No obvious publication bias was observed from funnel plot.

**Conclusion:** The current study suggested SMIBC had higher risk of CSM, OM and recurrence after RC, but lower risk of CSM after initial diagnosis.

## Introduction

Bladder cancer is the 12^th^ malignancy worldwide with around 550,000 patients were diagnosed of bladder cancer in 2018, making up 3% of all the malignancy, and around 200,000 patients died of it 1. When it comes to male patients, the ranking of bladder cancer rises (7^th^) by consisting of 4.5% of malignancy incidence worldwide 1. Urologically, the incidence of bladder cancer is the second worldwide and first in China 1, 2, causing a heavy socioeconomic burden. Pathologically, bladder cancer is categorized as non-muscle-invasive (NMIBC) and muscle-invasive (MIBC) according to the invasiveness of tumor in the wall of bladder. Over 70% patients are initially diagnosed as NMIBC and others are primarily diagnosed as MIBC (PMIBC) 3. However, up to 30% of NMIBC will progress to MIBC even after being given standard therapy 4. For those cases progressing to MIBC with previous NMIBC history, we define them as secondary MIBC (SMIBC). The standard therapy based on guideline for both PMIBC and SMIBC and neoadjuvant chemotherapy (NAC) with radical cystectomy (RC) 5.

Several studies have compared the survival prognosis of PMIBC with SMIBC and conflicting outcomes have been reported. For instance, Kotb 6 and his colleague found that SMIBC had better cancer-specific survival (CSS) but Breau 7 and Moschini 8 reported the risk of cancer-specific mortality (CSM) was lower in PMIBC, while May 9 revealed there was no significant difference between two groups. However, one of the key differences between those trials was the starting point of follow up-either from the initial diagnosis of bladder cancer or from radical cystectomy. As the development from NMIBC to SMIBC needs a certain interval, the comparison between PMIBC and SMIBC could be impacted. Therefore, it is unreasonable to pool all the reported outcomes in a single analysis, which was performed by a previous meta-analysis 10. In the current study, we propose a hypothesis that the outcomes of the prognosis comparison between PMIBC and SMIBC were different when the starting points of follow up are differentiated, and test it by including all published relevant comparative trials.

## Methods

Preferred Reporting Items for Systematic Reviews and Meta-Analyses (PRISMA) was followed to systematically search publications comparing PMIBC and SMIBC in PubMed, Embase, Cochrane Library and ClinicalTrial.gov. Literatures meeting the inclusion criteria were included: 1. Comparative trials comparing PMIBC and SMIBC; 2. Data regarding the cancer-specific mortality (CSM), overall mortality (OM) and recurrence-free survival (RFS) were provided in the fashion of hazard ratio (HR) with 95% confidence interval (CI). The exclusion criteria were: 1. None clinical trials such as review, meta-analysis and basic science; 2. Trials about MIBC but no comparison between PMIBC and SMIBC was performed; 3. Data not usable for the current study; 4. When overlapped data from the same cohort were published in different studies, the studies with smaller cohort size and shorter follow-up duration were excluded. Two members in our team independently searched the publications and extracted the data.

Data were extracted and differentiated based on the starting point of follow up (after initial diagnosis or after RC). The primary outcome was the pooled CSM and secondary outcomes were the OM and RFS. Pooled HR >1 indicated that SMIBC higher risk and HR <1 indicated that SMIBC had lower risk; Heterogeneity was calculated with I^2^. I^2^ >50% indicated that heterogeneity was high; Publication bias was assessed with funnel plot. When the funnel plot was symmetrical, publication bias was low; Newcastle-Ottawa scale was used to evaluate the study quality 11, 12, and studies with seven stars or more were ranked as high quality.

## Results

**Figure 1:**
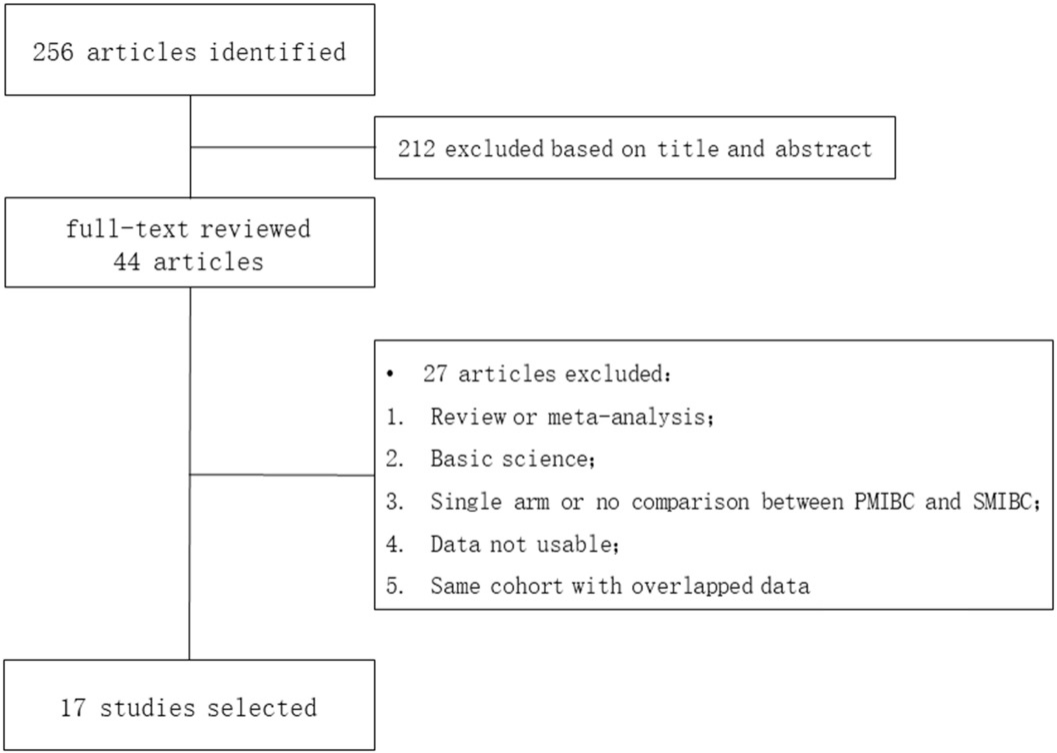
PRISMA flowchart of literature selection.

A total of 256 literatures were identified and 17 were eventually selected after subsequent title, abstract and full-text review (**Figure 1**) 6-9, 13-25. Published between 2002 to 2019, all those trials were retrospectively designed except May's and Breau's studies were prospective (**Table 1**). Patients in five trials were followed up after the initial diagnosis of bladder cancer and eleven were followed up after RC, while Hida's study provided outcomes both after initial diagnosis and RC. The mean follow-up duration ranged from 36 to 109 months. 5558 patients were involved with the mean age of 59.8 to 72.7 and the male-to-female ratio was close to 4:1 (4390/1124, data was not provided in Yiou's study). The study size was 3974 in PMIBC and 1584 in SMIBC (roughly 5:2).

**Figure 2:**
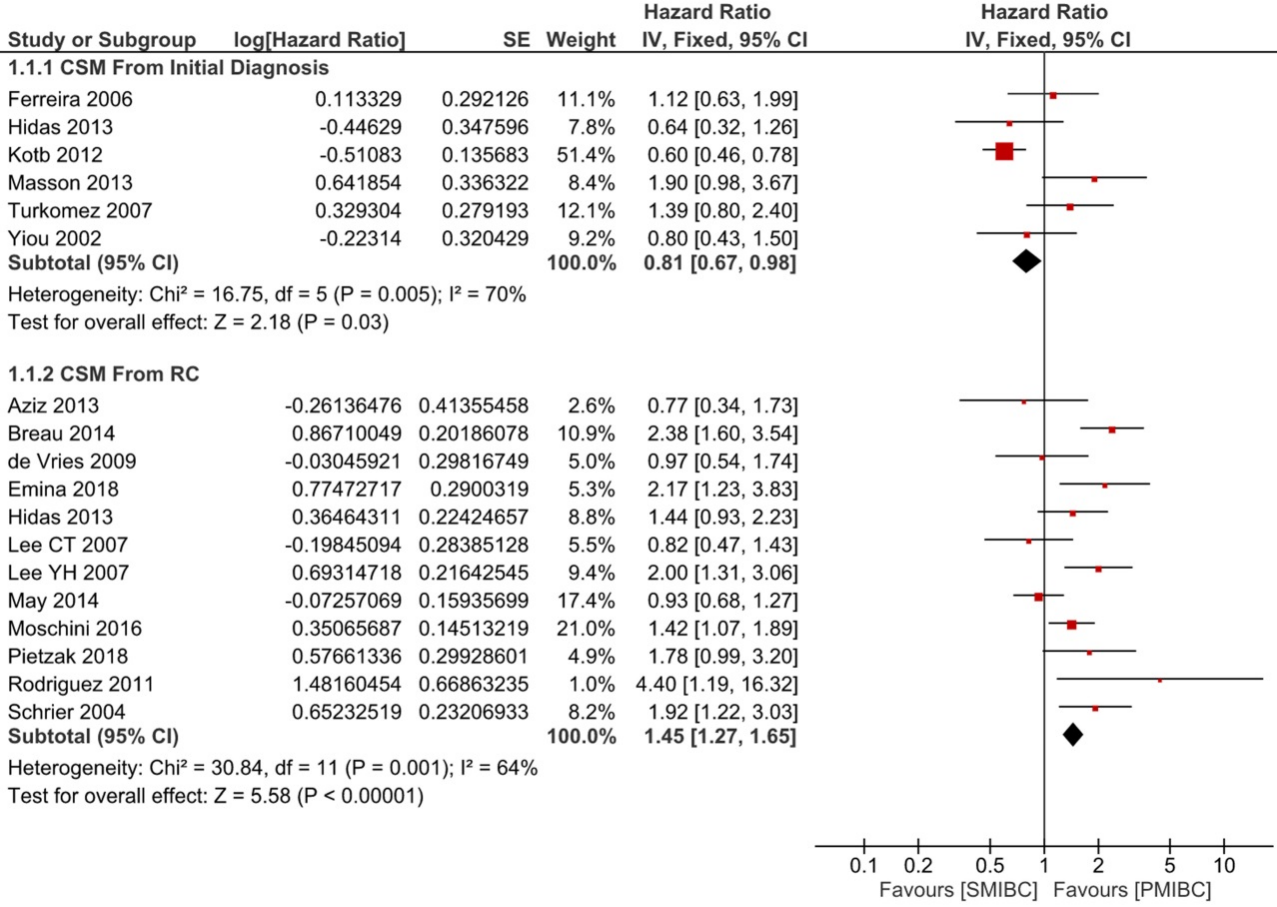
Comparison of SMIBC vs PMIBC for cancer-specific mortality after initial diagnosis or RC.

**Figure 3:**
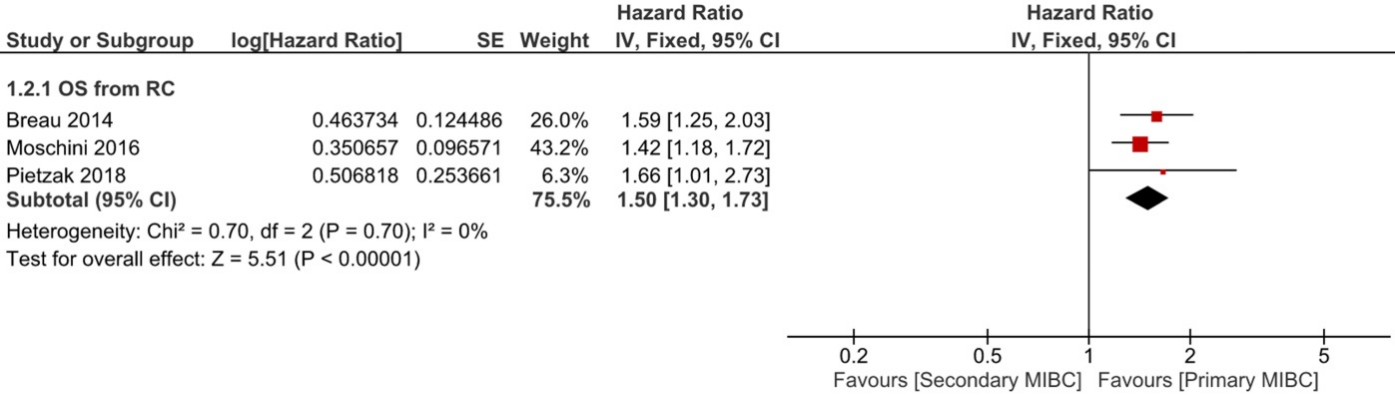
Comparison of SMIBC vs PMIBC for overall mortality after RC.

**Figure 4:**
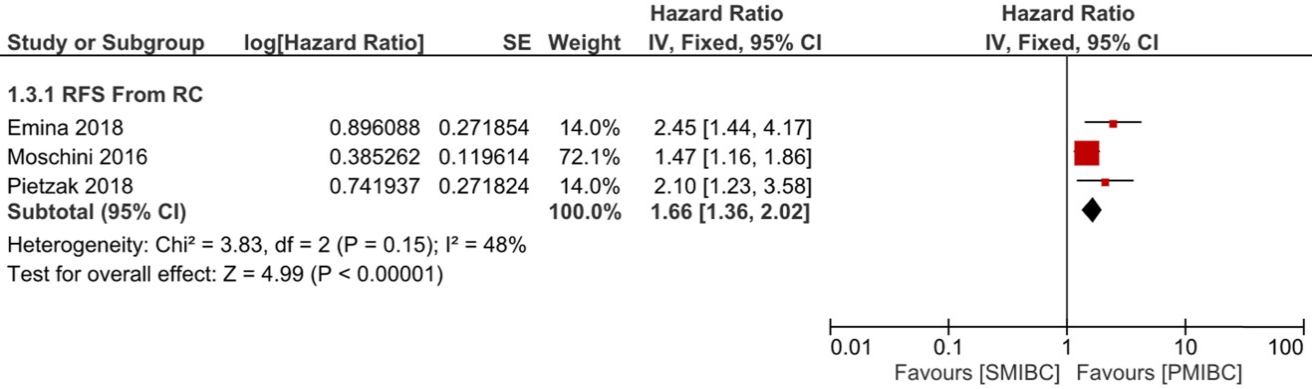
Comparison of SMIBC vs PMIBC for recurrence after RC.

Six trials with 1924 patients compared the risk of CSM after initial diagnosis of bladder cancer between SMIBC and PMIBC (**Figure 2**). The pooled HR indicated that SMIBC had lower risk of CSM after initial diagnosis (HR 0.81, 95%CI 0.67-0.98, *P=*0.03, I^2^=70%). However, pooled outcomes from twelve studies involving 3778 patients showed that the risk of CSM after RC was higher for SMIBC (HR 1.45, 95%CI 1.27-1.65, *P<*0.00001, I^2^=64%). OM after RC was reported in three trials (**Figure 3**) and pooled outcomes showed that it was higher in the SMIBC group (HR 1.50, 95%CI 1.30-1.73, *P<*0.00001, I^2^=0%). In terms of the risk of recurrence after RC (**Figure 4**), three trials reported the relevant outcomes and our analysis revealed that it was also higher for SMIBC (HR 1.66, 95%CI 1.36-2.02, *P<*0.00001, I^2^=48%).

All of the studies were ranked as high quality according to Newcastle-Ottawa Scale and no obvious publication bias were observed since the funnel plot was symmetric.

## Discussion

Research comparing PMIBC and SMIBC has been continuing for the last two decades. The first relevant study conducted by Vaidya reported that PMIBC had higher two-year disease-free survival than PMIBC (79% vs 49%) 26, but they did not perform further analysis to test if MIBC subtype was an independent risk factor of CSM, while the most recent study by Pietzak used Cox multivariate regression to find SMIBC has lower risk of CSM (HR 1.66, 95 CI 1.01-2.73, *P* = 0.048) and recurrence (HR 2.10, 95 CI 1.23-3.57, *P* = 0.007) 25. Between the publications of those two trials, other studies have been published but controversies still remain. As mentioned above, Kotb reported that SMIBC had lower risk of CSM (HR 0.60, 95 CI 0.47-0.80, *P* = 0.0003) 6, while May claimed that no significant difference regarding CSM was observed between two groups (HR 0.93, 95 CI 0.68-1.27, *P* = 0.637) 9. Notably, outcomes were not reported uniformly in those trials, nor did the subsequent meta-analysis by Ge and his colleague. In Ge's study, pooled HR was reported indicating a higher risk of CSM for SMIBC group 10, but outcomes from the initial diagnosis and from RC were both included in the same analysis. Therefore, we performed the current meta-analysis and confirmed the risk of CSM, OM and recurrence was higher for SMIBC group only after RC. However, in the long run from the initial diagnosis of bladder cancer, SMIBC had a lower risk of CSM compared with PMIBC.

A reasonable speculation for our finding was SMIBC had relatively longer duration of disease development, which was partially supported the one year longer follow up in the SMIBC group compared with PMIBC in Hidas's (52.6 months vs 40.1 months) and Turkolmez's studies (90.3 months vs 77.8 months) 18, 23.Therefore, the relatively longer survival time after initial diagnosis of SMIBC eventually led to a lower risk of CSM. However, due to the progressive nature, SMIBC could led to a worse prognosis once it progressed to muscle-invasive. Moreover, Pietzak claimed that ERCC2 mutation is more common in PMIBC 25, which was previously reported to be able to increase the sensitivity to chemotherapy and improve the prognosis of MIBC 27-29. However, advance regarding the molecular mechanism behind PMIBC and SMIBC is limited so far and future researches are further required.

The administration of NAC has become a standard procedure for MIBC before RC, showing encouraging effect in several studies 30-32. In the selected trials, Kayama supported NAC could lead to better CSS for PMIBC while Pietzak claimed only the risk of recurrence decreased after using NAC. In terms of SMIBC, NAC was found to correlate with higher risk of CSM, OM and recurrence, which might be caused by the delay of RC. However, we could not perform a subgroup analysis according to the usage of NAC or not because of the limited number of related studies.

One of the advantages of the current study is the differentiation of different starting point of follow up. Compared with Ge's study, our study confirmed the risk of CSM was higher for SMIBC after RC, but we also found that SMIBC had better prognosis when follow up started from the initial diagnosis of bladder cancer, which actually address the necessity of differentiation of follow up. Furthermore, our study included more trials and not only pooled the outcomes of CSM but also OM and recurrence-free survival. There are two implications of our findings: First, SMIBC was proved to be more lethal than PMIBC after NMIBC progressed to muscle-invasiveness. In other word, SMIBC has a more progressive nature as long as muscle-invasiveness was confirmed, which also implied that physicians should pay special attention to the disease progression of SMIBC patients and give them more individualized medical care; Second, our outcomes also partly resolve the controversies between the prognosis comparison between SMIBC and PMIBC, which was most likely caused by different starting point of follow up.

However, our study is not without drawbacks. The first drawback of the current study is the high heterogeneity in the analyses. As the prognosis of MIBC is impacted by multiple factors such as age, tumor size and lymphovascular invasion, but the variables in the multivariate regression is not consistent among the selected trials. Moreover, this also suggests the necessity of pooled outcomes. Another drawback is the absent analyses of postoperative complications and life quality, which requires more focus of future trials.

## Conclusion

The pooled outcomes of the current meta-analysis suggested that it is necessary to differentiate the starting point of follow up when comparing the prognosis of SMIBC and PMIBC by showing that SMIBC had higher risk of CSM, OM and recurrence after RC but lower risk of CSM after initial diagnosis of bladder cancer.

## Figures and Tables

**Figure 5:**
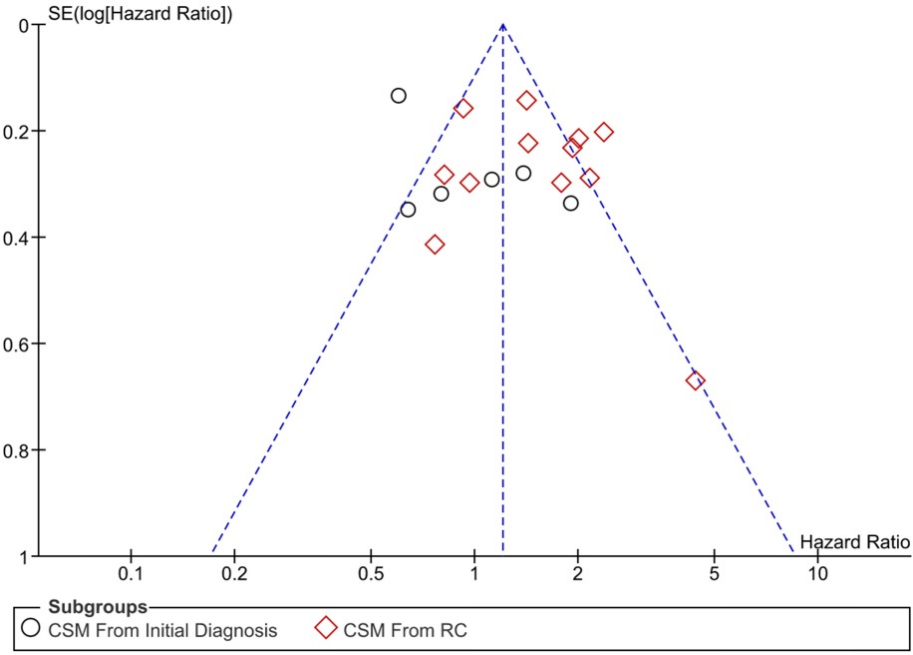
Funnel plot of publication bias.

**Table 1:** Characteristics of included studies

Study	Year	Country	Design	Size		Gender (m/f)		Age (P/S)	Starting point	Follow-up duration (P/S)	Study Quality
				PMIBC	SMIBC	PMIBC	SMIBC				
Yiou	2002	France	R	43	12	NA	NA	62/66	Initial	49/55.3	8
Schrier	2004	Netherland	R	89	74	65/24	60/14	63.3/68.5	RC	NA	8
Lee YH	2007	South Korea	R	173	50	154/19	46/4	<60 : 68/15 >60 : 105/35	RC	45	8
Turkomez	2007	Turkey	R	109	45	134/20		59.8/60.3	Initial	77.8/90.3	9
Lee CT	2007	US	R	169	70	127/42	55/15	65/69	RC	40/33	8
Ferreira	2007	Brazil	R	185	57	145/40	47/10	63.7/65.3	Initial	NA	8
de Vries	2009	Netherland	R	134	54	103/31	41/13	61/63	RC	41	8
Rodriguez	2011	Spain	R	72	69	116/25		NA	RC	42.5	8
Kotb	2012	Canada	R	785	365	623/161	291/74	66.7/67.2	Initial	NA	9
Hidas	2013	Israel	R	104	40	79/25	33/7	72.7/69.3	Initial + RC	44	8
Masson	2013	France	R	155	24	146/21	20/4	66.8/68	Initial	36	-
Aziz	2013	Germany	P	125	25	97/28	24/1	69/71	RC	46	9
May	2014	Germany	P	399	122	296/103	92/30	64.1/68.7	RC	65	9
Breau	2014	Canada	R	481	190	366/115	146/44	67.6/66.5	RC	NA	9
Moschini	2016	Italy	R	475	293	391/84	250/43	68/67	RC	109	9
Emina	2018	Japan	R	231	51	188/43	40/11	70.2/71.6	RC	NA	9
Pietzak*	2019	US	R	245	43	181/64	34/9	65/68	RC	48	9

*Only data of patients received neoadjuvant therapy was included in analysis. Abbreviations: R, retrospective; P, prospective.
